# Genome-Wide Identification and Characterization of Toll-like Receptors (TLRs) in *Diaphorina citri* and Their Expression Patterns Induced by the Endophyte *Beauveria bassiana*

**DOI:** 10.3390/jof8080888

**Published:** 2022-08-22

**Authors:** Luis Carlos Ramos Aguila, Hafiza Javaira Ashraf, Jessica Paola Sánchez Moreano, Komivi Senyo Akutse, Bamisope Steve Bamisile, Liuyang Lu, Xiaofang Li, Jingyi Lin, Qing Wu, Liande Wang

**Affiliations:** 1 State Key Laboratory of Ecological Pest Control for Fujian and Taiwan Crops, Key Laboratory of Biopesticide and Biochemistry, MOE, College of Plant Protection, Fujian Agriculture and Forestry University, Fuzhou 350002, China; 2 Key Laboratory of Vegetation Restoration and Management of Degraded Ecosystems, South China Botanical Garden, Chinese Academy of Sciences, Guangzhou 510650, China; 3 Institute of Horticultural Biotechnology, Fujian Agriculture and Forestry University, Fuzhou 350002, China; 4 International Centre of Insect Physiology and Ecology (*icipe*), Nairobi P.O. Box 30772-00100, Kenya; 5 Department of Entomology, College of Plant Protection, South China Agricultural University, Guangzhou 510642, China

**Keywords:** entomopathogenic fungi, Toll-like receptor, pathogens, immunity, endophytes

## Abstract

Toll-like receptors (TLRs) are pathogen recognition receptors (PRRs), which play key roles in helping the host immune system fight pathogen invasions. Systematic information on TLRs at the genome-wide level and expression profiling in response to endophytic colonization is very important to understand their functions but is currently lacking in this field. Here, a total of two TLR genes were identified and characterized in *Diaphorina citri*. The TLR genes of *D. citri* were clustered into five families according to the phylogenetic analysis of different species’ TLRs. The domain organization analyses suggested that the TLRs were constituted of three important parts: a leucine-rich repeat (LRR) domain, a transmembrane region (TR) and a Toll/interleukin-1 receptor (TIR) domain. The mRNA expression levels of the two *TLR* genes (*DcTOLL* and *DcTLR7*) were highly regulated in both nymphs and adults of *D. citri*. These results elucidated the potentiated TLR gene expression in response to endophytically colonized plants. Furthermore, the 3D structures of the TIR domain were highly conserved during evolution. Collectively, these findings elucidate the crucial roles of TLRs in the immune response of *D. citri* to entomopathogens systematically established as endophytes, and provide fundamental knowledge for further understanding of the innate immunity of *D. citri*.

## 1. Introduction

Multicellular organisms have an inherent immune system that helps in preventing the invasion of pathogens; as soon as the host body detects pathogens, it triggers innate immune responses to block the invaders and then primes the body’s adaptive immunity against them [1,2]. Arthropods mainly depend on the innate immune system to face infection by pathogens. According to Ligoxygakis [3] there are basically two signaling pathways involved in insect immunity (Toll (or Toll-like receptor, TLR) pathway and immune deficiency (Imd) pathway). The Toll/TLR pathway is involved in both development and immunity, while the Imd pathway is only involved in immunity [4].

The TLR family consists of highly conserved pattern recognition receptors (PRRs), that recognize pathogen-associated molecular patterns (PAMPs). PAMPs are highly conserved small molecules associated with pathogens, and these molecules include lipoprotein, lipopolysaccharide, mannose, lipoteichoic acid, peptidoglycan and nucleic acid molecular structures [5,6]. As a result of the host PRR’s interaction with pathogens, an immune response is initiated to eliminate them or overcome their infection [7]. TLRs are a class of PRRs that has two main components, an extracellular leucine-rich repeat (LRR) region and an intracellular Toll/interleukin (IL)-1 receptor (TIR) domain. The function of the extracellular LRR region is to identify pathogens; meanwhile, the intracellular TIR domain responds to downstream signaling. Based on this structural composition, LRRs link extracellular signals to intracellular specific gene expressions [8,9]. To date, these family members have been studied at the genome-wide level in several animals. For example, 10 TLRs have been identified in humans, named TLR1-10, while 12 TLRs have been identified in mice, named TLR1-12 [10,11]; among them, TLR1,2 and TLR4,6 are positioned in the plasma membrane and the function they perform is the recognition of extracellular pathogens [12,13]. The TLR2 receptor recognizes *Propionibacterium acnes* to induce inflammatory cytokines [14]. The TLR5 receptor recognizes and binds to the flagellin protein of some Gram-positive and Gram-negative bacteria, and triggers the downstream innate immune response [15]. TLR3,7,8 locate within endosomes, recognize viral DNA or RNA that has invaded the cytoplasm, and activate the antiviral immune response [16]. TLR roles in antimicrobial immune responses have also been reported [17]. Six TLRs have been identified in *Musca domestica* (Diptera: Muscidae) named TLR1-6, and they play crucial roles in the immunity of the housefly [18]. Several studies have also been carried out in crustaceous species [19,20,21,22]. However, TLR studies at the genome-wide level are currently lacking in the insect lineage.

*Diaphorina citri* is a major emerging pest of citrus production worldwide, because it is a vector for *Candidatus* Liberibacter asiaticus, the causal agent of Huanglongbing, also known as citrus greening, the most damaging disease in citriculture [23,24]. TLRs are very important elements of innate immunity. However, the TLRs and their underlying functional mechanisms in *D. citri* have not been exhaustively addressed to date. Using genome-wide analysis, we can gain a systematic understanding of a particular gene family, including member classification, phylogenetics, gene expression, and molecular evolution [25]. In the present study, we aimed to (i) characterize TLR genes in *D. citri* through genome-wide identification; and (ii) test the hypothesis that *C. sinensis* seedlings endophytically colonized by *B. bassiana* lead to changes in TLR gene expression in *D. citri* when feeding on them. Two TLRs were identified in the *D. citri* genome, and their phylogenetic relationship with other TLRs from different species were analyzed. Furthermore, the expression profiles of TLR family members in *D. citri* fed on endophytically colonized *C. sinensis* seedlings were studied and the expression analyzed in nymphs and adults. Besides, structural alignments showed that the 3-dimensional structures of TIR domains were highly conserved among the two TLRs identified. Moreover, the protein-protein interaction (PPI) network analysis of *D. citri* TLRs suggested some potential interactors in innate immune signaling. This study analyzed the outcomes of interaction between *D. citri*-endophyte-*C. sinensis* in relation to the immune responses and provides a genomic foundation for a better understanding of the effects of entomopathogens in *D. citri* innate immunity.

## 2. Materials and Methods

### 2.1. Plant Material, Source of Fungal, Conidia Suspension Preparation and Inoculation

Seedlings of *C. sinensis* used in this experiment were raised from surface-sterilized seeds (soaked for 3 min in 75% ethanol, followed by 3 min in 2% sodium hypochorite; then they were rinsed three times in sterile distilled water). The seeds were planted in germination trays (40 × 30 × 8 cm) containing sterile potting compost (the potting compost was sterilized at 120 °C for two hours in an autoclave), and once the seedlings had three true leaves, they were transplanted into plastic pots (8 cm high × 7.5 cm diameter) containing sterile planting compost. Transplants were then kept in a glass room at 25 ± 2 °C, 70 ± 5% RH, and 12:12 (L:D) photoperiod.

*Beauveria bassiana* strain 16 (BB-16) was obtained from the Insect Ecology and Biological Control Laboratory at Fujian Agriculture and Forestry University, Fuzhou, China. We identified the strain using morphological descriptions based on the Humber [26,27] keys. The ITS region of nuclear rRNA of the isolate (GenBank accession number MG844431) was sequenced and used along with the morphological features to confirm the identity of the isolate. The isolate BB-16 was cultured on Potato Dextrose Agar (PDA) culture media (Qingdao Hope Bio-technology Co., Ltd. Qingdao, China) in Petri dishes (90 × 15 mm) at constant temperature of 25 ± 2 °C, 65–75% relative humidity for 18 days in complete darkness. Conidia were harvested under sterile conditions by gently scraping conidia from the culture surface using a spatula and suspending them in sterile distilled water (SDW) containing Tween 80 (0.01%) to emulsify. The suspension was homogenized by vortexing the suspension for 4 min, and then the hyphal debris was removed by filtering with a sterile syringe and cotton wool. Finally, the conidial concentration was adjusted to 1 × 10^8^ conidia mL^−1^ under the microscope using a Neubauer hemocytometer. Prior to inoculation, 100 µL of the first serial dilution was planted on 2.5% water agar and then incubated at 25 °C for 24 h to determine the percentage of conidia viability. Germinated conidia were counted to determine conidia viability. A conidium was considered viable when its germ tubes were longer than half its diameter. Inoculation with conidial suspensions was limited to those with germination rates of 90% or higher.

The inoculation was done approximately 40 days post-transplanting date. Seedlings within an average height of 12 cm and at least 6–7 true leaves were selected for inoculation. This was done to ensure an accurate count of eggs, nymphs and adults. Fifty seedlings each were foliar-sprayed with an average of 5 mL of 1 × 10^8^ conidial mL^−1^ suspension of *B. bassiana*, while the control seedlings were sprayed with sterile distilled water containing 0.01% Tween 80 solution.

### 2.2. Colonization Assessment

Plant colonization assessment was determined/conducted throughout re-isolation of endophytic *B. bassiana* BB-16 from inoculated plants at 7 and 28 days post foliar inoculation (dpfi) using the methodology described by Greenfield, et al. [28]. Ten inoculated plants were sampled on each sampling day. Selected leaves were cut into 2 cm^2^ and then surface-sterilized by immersing in 70% ethanol for 1 min and in 1.5% sodium hypochlorite for 1 min and finally rinsed three times in sterile water.

The last rinsing water was plated to assess the effectiveness of surface sterilization of plant materials, and plate imprinting was also conducted [29]. Afterward, leaf segments were allowed to surface-dry on sterile paper towels in a laminar flow hood. Since the sterilization process might have eradicated the endophytes in the outer edges of the leaf segments, the outer parts were removeds using a blade disinfected with 75% ethanol between cuts. Five small tissue pieces (8 mm wide) were placed on freshly prepared PDA plates amended with Streptomycin sulfate and chloramphenicol at 1.25 g L^−1^ to suppress bacterial contamination/growth, sealed with parafilm and stored in the dark at 25 °C; plates were examined at two day intervals to observe and record fungal growth. Leaf tissues that disclosed fungal emerging colonies were isolated and transferred into new PDA plates. Furthermore, to confirm that the emerging endophytes were similar to the inoculated fungal isolate, fungal outgrowth from plated leaf tissueswere morphologically identified by comparing the mycelia, colony morphology, and growth pattern with the mother culture and by microscopic observation and taxonomy keys in line with Humber [26,27], viewing the conidia and conidiophores using a light microscope (Model CX23LEDRFS1C, Olympus Corporation, Tokyo, Japan).

Colonization percentage was calculated with reference to Petrini and Fisher [30] formula as:

$$\mathrm{Colonization}\;\%=\frac{\mathrm{Number}\;\mathrm{of}\;\mathrm{leaf}\;\mathrm{segments}\;\mathrm{showing}\;\mathrm{fungal}\;\mathrm{outgrowth}}{\mathrm{Total}\;\mathrm{number}\;\mathrm{of}\;\mathrm{incubated}\;\mathrm{leaf}\;\mathrm{segments}\;}\times 100$$

Additionally, leaf colonization by *B. bassiana* BB-16 at 7 and 28 dpfi was determined using PCR-based molecular techniques. Leaves from 5 non-treated plants and treated plants were randomly selected and sterilized using the same procedure as described above. For the DNA extraction 0.5 g of leaves from each group were ground with liquid nitrogen and the genomic DNA of *B. bassiana* BB-16 was extracted using TIANGEN^®^ Plant Genomic DNA Kit [Tiangen Biotech (Beijing) Co., Ltd., Beijing, China] following the protocol provided by the manufacturer. ITS region of the rDNA was amplified using universal fungal primers ITS4-F (5′-TCC TCC GCT TAT TGA TAT GC-3′) and ITS5-R (5′-GGA AGT AAA AGT CGT AAC AAG G-3′) [31]. PCR amplification was performed with a thermal cycler (Applied Biosystems 2720 Thermal Cycler, Foster city, CA, USA), as follows: an initial denaturation step consisting of 5 min at 95 °C; 35 cycles of 30 s at 95 °C, 1 min at 57 °C, 1 min at 72 °C, and a final extension of 6 min at 72 °C followed by a 4 °C soak. The amplified PCR products were visualized on 1% agarose gel. Purified PCR products were sent to a commercial facility (BioSune Pvt, Ltd. Fuzhou, China) for sequencing. Sequence data were cleaned and subjected to BLAST analysis on the NCBI (National Center for Biotechnology Information) web tool to validate the characteristics of the amplified sequences. The fungus was identified as *B. bassiana*, since the amplified sequences information shown complete congruence between BB-16 and the fungus recovered as an endophyte (97.87%).

### 2.3. Diaphorina citri Assays

*Citrus sinensis* seedlings endophytically colonized by *B. bassiana* and free-endophytes were placed each in 20 rearing cylinder bottles (20 cm tall, 8 cm diameter); at the bottom center, a circular opening (6 cm diameter) was made and covered with a fine mesh for aeration. Then, one newly emerged adult female and male of *D. citri* (2 days old) were collected from the stock population, enclosed in the rearing bottle and removed after a 24 h. After this, the newly emerged adult females and males (2 days old) from endophytically colonized plants and uncolonized plants were paired and subsequently enclosed in individual rearing bottles with a freshly treated *C. sinensis* seedling respectively, and allowed oviposition for 24 h; then adults were removed. Later nymphs and adults of *D. citri* were sampled to carry out the expression profiles of TLRs family in *D. citri* fed on endophytically colonized *C. sinensis* and control, and the analysis was done with the first generation.

### 2.4. TLRs Identification and Domain Organization Analysis

The protein sequence data of *D. citri* were downloaded from the *D. citri* Genome and Transcriptome database (https://citrusgreening.org/organism/Diaphorina_citri/genome accessed on 26 September 2021). The Hidden Markow Model (HMM) profiles of the LRR (PF00560) and TIR (PF01582) were obtained from the Pfam database (http://pfam.xfam.org/ accessed on 28 September 2021) and employed to search the TLRs genes in the *D. citri* genome. The HMM profiles of LRR (PF01582) repeats and TIR (PF00560) domain. Proteins containing LRR repeats and TIR domains were identified by the HMMscan (http://www.ebi.ac.uk/Tools/hmmer/search/hmmscan accessed on 29 September 2021). As a supporting test for the LRR and TIR domain, the candidates were checked manually in the SMART (http://smart.embl-heidelberg.de/ accessed on 29 September 2021) and PROSITE (http://prosit.expasy.org/index.html accessed on 29 September 2021) online software, and then the redundant sequences were removed. The subcellular locations of *DcTLRs* were predicted in the Wolf PSORT online server (https://wolfpsort.hgc.jp accessed on 8 November 2021). The molecular weights (Mw) and isoelectric point (pl) values were calculated by ExPASy online server (https://web.expasy.org/compute_pi/ accessed on 10 November 2021).

### 2.5. Phylogenetic Analysis

The two amino acid sequences *DcTOLL* and *DcTLR7* identified in *D. citri* and 68 TLR from seven different species, i.e., the available TLR genes in vertebrates including *Homo sapiens* (Primates: Hominidae), *Mus musculus* (Rodentia: Muridae) *Bombyx mori* (Lepidoptera: Bombycidae), *Musca domestica* (Diptera: Muscidae), *Acyrthosiphon pisum* (Hemiptera: Aphididae), *Drosophila melanogaster* (Diptera: Drosophilidae) and *Apis mellifera* (Hymenoptera: Apidae) were composed from the NCBI database and published papers (Table S1). Then sequences were imported into MEGA X software and aligned by ClustalW. The phylogenetic tree was constructed using the Neighbor-Joining (NJ) algorithm method [32] with JTT model, pairwise gap deletion and 1000 bootstraps.

### 2.6. TLRs Domain Organization, Chromosome Location and Structural Analysis TIR Domain

The predicted protein domain architecture of the two Toll-like receptor genes (*DcTOLL* and *DcTLR7*) was determined by the SMART server with the default parameters; further, the results were confirmed by LRR finder and TMHMM server. The chromosomal number, chromosomal locations of *DcTOLL* and *DcTLR7* start and end positions, and the chromosomal sequence length were determined by annotations in the genome database at (https://citrusgreening.org/organism/Diaphorina_citri/genome accessed on 5 October 2021). To show the location of the *DcTLRs* in the *D. citri* genome, we manually mapped the two *DcTLRs* positions along the two *D. citri* chromosomes using TBtools Toolkit software [33].

Sequence alignment of the amino acid sequences of the TIR domains of the insect TLRs was performed through the *Clustal*W method, and the functional boxes were identified through literature review analysis. After the identification of these conserved boxes, the online site Multiple EM for Motif Elicitation, v.11.2 (MEME) (http://memesuite.org/ accessed on 10 October 2021) was implemented to finish sequence logos analysis in the complete amino acid sequences of different insect TLRs.

### 2.7. RNA Extraction, cDNA Synthesis, qRT-PCR Reactions and Expression Analysis

To analyze the nymph and adult-specific expression patterns of *DcTLR* genes in *D. citri* fed on endophyte-free plants and endophytically colonized plants, the total RNA from *D. citri* nymphs and adults were extracted using TIANGEN^®^ DNA/RNA Isolation Kit [Tiangen Biotech (Beijing) Co., Ltd.] following the protocol provided by the manufacture. TIANGEN^®^ Fastking gDNA Dispelling RT SuperMix [Tiangen Biotech (Beijing) Co., Ltd.] was used to synthesize first-strand cDNA following the protocol of the manufacture. The qRT-PCR reactions were performed with SuperReal PreMix Plus (SYBR Green) [Tiangen Biotech (Beijing) Co., Ltd.] according to the manufacturer’s instructions in the BIO-RAD CFX96^TM^ Real-Time System; three technical repeats and three biological replicates were conducted for each treatment. The *ACT1* gene was used as the internal control [34]. The primer sequences of *ACT1* and *DcTLRs* are listed in Table S2; first chain cDNA synthesis volume and real-time qPCR reaction volume are listed in Tables S3 and S4, respectively. The relative gene expression was calculated using the 2^−ΔΔCt^ method. All values were performed with GraphPad Prism 9 (GraphPad Software Inc., La Jolla, CA, USA) and represented as the mean ± SEM. The statistical differences between groups were determined using Student’s *t*-test.

### 2.8. Primary and 3-Dimensional Structural Analysis

The amino acid sequence of the TIR domains in 2 *DcTLR* proteins was aligned using the web tool Clustal Omega (http://www.ebi.ac.uk/Tools/msa/clustalo/ accessed on 3 February 2022) and its visualization was carried out in Jalview software (version 2), Waterhouse, Dundee, UK [35]. As the 3-Dimensional (3D) structures of *D. citri* TIR domains have not been previously represented, the online server SWISS-MODEL (https://swissmodel.expasy.org/interactive accessed on 3 February 2022) was adopted to obtain the 3D models of the 2 *DcTLR* through homology modeling; then, the PDB files of the 3D models were downloaded. Further, structural alignments and all 3D structural figures were generated by PyMOL software (version 2.0) Warren Lyford DeLano, CA, USA.

### 2.9. Protein-Protein Interaction Network Study

The protein-protein interaction (PPI) data of *D. citri* TLRs were obtained from the String server (https://string-db.org/ accessed on 5 February 2022). Briefly, the query search protein by sequence was used to find the PPI of *DcTLR*. The brightness of nodes was represented according to their degree values. The computer software Cytoscape (version 3.7.2) was employed to construct the interaction network maps.

## 3. Results

### 3.1. Endophytic Colonization Assessment

**Figure 1:**
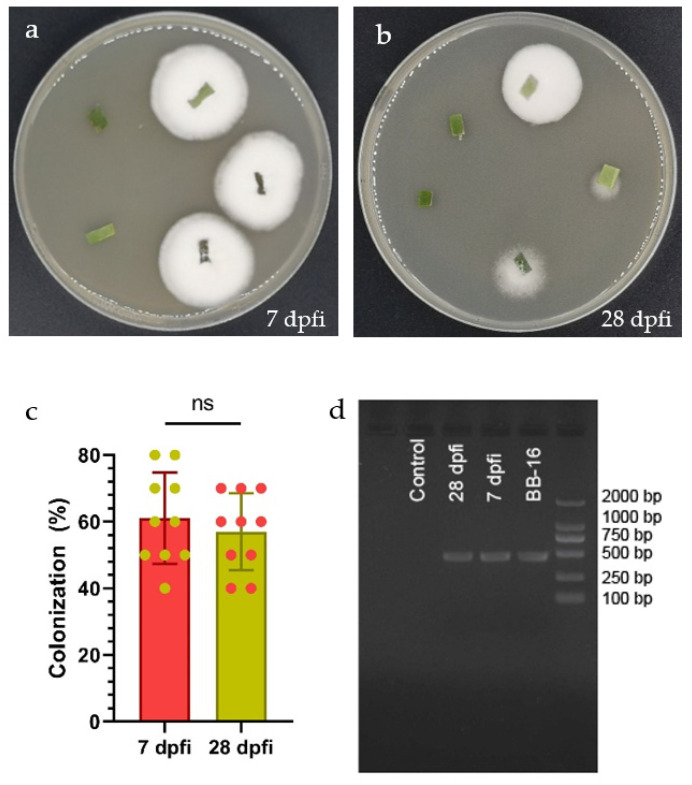
(**a**,**b**) *Beauveria bassiana* re-isolated from leaves at 7 and 28 dpfi, respectively. (**c**) Mean (+SE) percent colonization of *Citrus sinensis* leaves by *Beauveria bassiana* BB-16 at 7 and 28 dpfi; data were analyzed using *t*-test at *p* < 0.0001. (**d**) Detection of *Beauveria bassiana* BB-16 in newly emerged leaves from inoculated plants sampled at 7 and 28 dpfi using PCR assay.

*Beauveria bassiana* BB-16 colonization of *C. sinensis* leaves was detected at 7 and 28 days post foliar inoculation (dpfi) by the culture-based technique (Figure 1a,b). *B. bassiana* BB-16 was successfully re-isolated from inoculated leaves as can be seen from the white dense mycelial outgrowth from leaf segments obtained from foliar inoculated plants and planted on PDA culture media. The fungal outgrowth was further confirmed as *B. bassiana* by microscopy observation. No significant differences were observed in the colonization percentage of inoculated plants at 7 and 28 dpfi (Figure 1c). In addition, colonization of *C. sinensis* leaves by *B. bassiana* BB-16 was detected using PCR at 7 and 28 dpfi (Figure 1d). The PCR assays confirmed the endophytic colonization, while no band was obtained from the control plant.

### 3.2. Identification and Characterization of TLR Genes in D. citri

Results from the identification revealed a total of two non-redundant TLR family members in the *D. citri* genome, and these genes were named *DcTOLL* and *DcTLR7* based on the phylogenetic analysis. The detailed information on the identified genes and their coding proteins were listed in Table 1. The predicted size of *DcTOLL* and *DcTLR7* were 374 and 1323 aa, respectively, with ORFs ranging from 1125 bp (*DcTOLL*) to 3972 bp (*DcTLR7*). The isoelectric points of the two *DcTLRs* were similar and close to neutral, varying from 6.01 (*DcTOLL*) and 6.09 (*DcTLR7*). The molecular weight for *DcTOLL* and *DcTLR7* were 43,503.94 and 150,543.47 KDa, respectively. All studied *DcTLR* genes showed GRAVY below zero, which indicated that these two proteins were hydrophilic. Subcellular localization of the proteins was predicted to be in the plasma membrane (Table 1).

### 3.3. Phylogenetic Analysis of Insect TLR Genes

**Figure 2:**
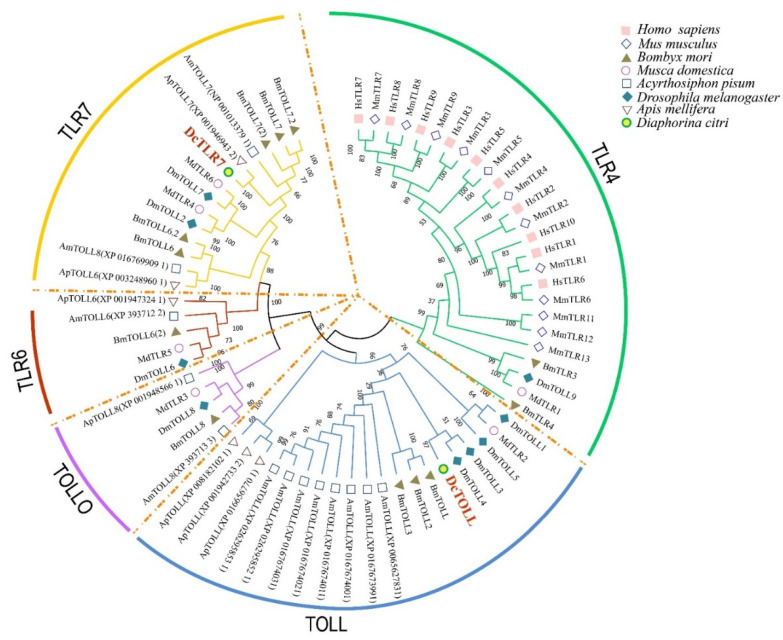
Phylogenetic tree with full-length amino acid sequences of *Diaphorina citri*, *Apis melifera*, *Drosophila melanogaster*, *Acyrthosiphon pisum*, *Musca domestica*, *Bombyx mori*, *Mus* musculus and Homo sapiens TLR proteins. The maximum likelihood method using MEGA X software with 1000 bootstrap replicates was used to construct the phylogenetic tree. The tree could be divided into 5 major clusters, and each cluster was named according to the dominant proteins and marked with different colors.

Results showed that the species used in the analysis could be divided into five groups, including TOLL, TOLLO, TLR4, TLR6 and TLR7. TLR4 was the largest group, including 26 TLRs; TOLL was the second largest group with 20 TLRs. The two *DcTLRs* were divided into the TLR7 and TOLL groups (Figure 1). As shown in Figure 1 in respective colors, the two *DcTLRs* genes shared the closest evolutionary relationship with the corresponding TLRs from *M. domestica*, *B. mori*, *A. pisum*, *D. melanogaster* and *A. mellifera*, indicating an ancestral relationship among them (Figure 2).

### 3.4. Domain Organization and Chromosome Location Analysis

**Figure 3:**
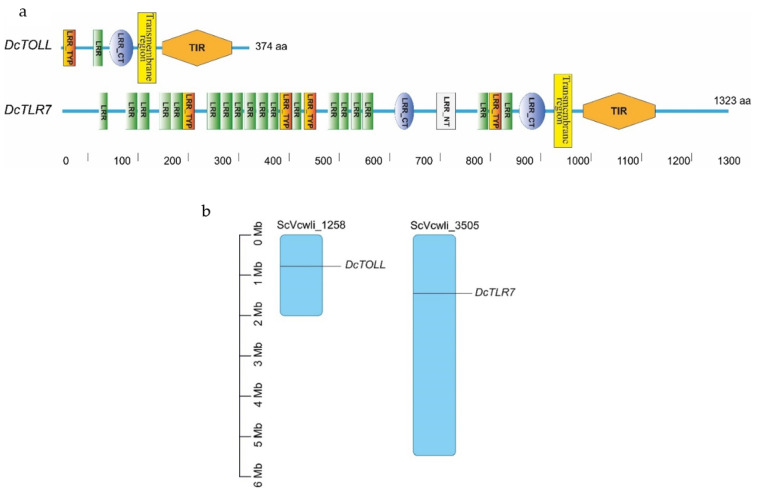
(**a**) Schematic representation of the domain organization of *DcTLRs;* different colors and shapes represent the different domains and regions. (**b**) Chromosomal localization of *Diaphorina citri* TLRs. The size of a chromosome is indicated by its relative length.

In *D. citri* TLRs, the transmembrane region and LRR repeats were detected in the two TLRs. *DcTOLL* had one LRR repeat; meanwhile, there were 18 LRR repeats in *DcTLR7,* which has the longer ectodomain of the two TLRs. The longer the ectodomain, the more LRR repeats. LRR carboxyl-terminal domain (LRR_CT) was present in the two TLRs; however, *DcTOLL* had one LRR_CT and *DcTLT7* had two LRR_CT. LRR amino-terminal domain (LRR_NT) was present only in *DcTLR7*, while no LRR_NT was detected in *DcTOLL*, which may be related to its short LRR region (Figure 3a). Leucine-rich repeats, typical subfamily (LRR_TYP), were present in the two TLRs; however, *DcTOLL* had one LRR_TYP and *DcTLT7* had four LRR_TYP.

Results show that *DcTLR7* and *DcTOLL* genes were unevenly distributed across the genome, and *DcTOLL* was located on chromosome ScVcwli_1258; meanwhile, *DcTLR7* was located on chromosome ScVcwli_3505 (Figure 3b). In addition, the results also showed that there is no tandem duplication since *DcTLR7* and *DcTOLL* were located in different chromosomes.

### 3.5. Structural Analysis of D. citri TIR Domain

**Figure 4:**
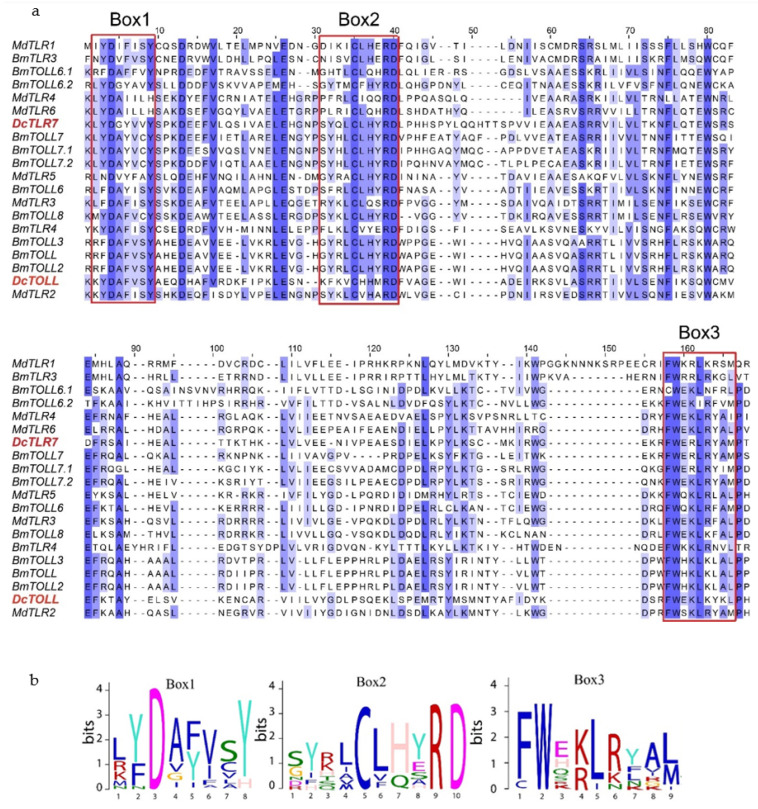
(**a**) Sequence alignment of TIR domains from *Diaphorina citri*, *Bombyx mori* and *Musca domestica*; sequences in a red square are Box1, 2 and 3, respectively. (**b**) Sequence logos of Box1, 2 and 3 from the sequence alignment.

Results from the amino acid sequences alignment of the insect TLRs revealed the TIR domains were characterized by three highly conserved regions (Boxes) (Figure 4a). Box1 was positioned at the N terminal of the TIR domain and contained a highly conserved aspartate (D). Box2 was positioned next to Box1 and contained highly conserved cysteine (C), arginine (R) and aspartate (D) residues. Box3 was positioned at the C terminal of the TIR domain and contained highly conserved phenylalanine (F), tryptophan (W) and leucine (L) residues (Figure 4b).

### 3.6. The 3D Structure of TIR Domain in Diaphorina citri

**Figure 5:**
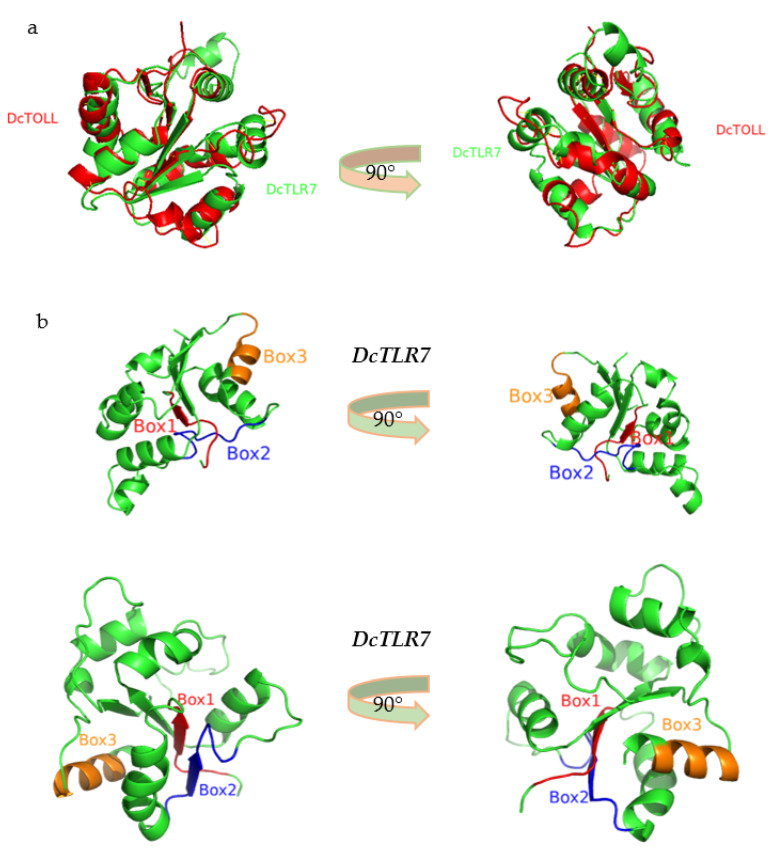
(**a**) Structural alignments of two TIR domains models from *Diaphorina citri.* (**b**) The 3D model of the TIR domain in *DcTLR7* and *DcTOLL.* Box 1, Box 2 and Box 3 are labeled in different colors.

The predicted 3D models were generated based on the amino acid sequence of the TIR domains of *DcTLR7* and *DcTOLL,* and structural alignments exhibited that these TIR domains were structurally conserved (Figure 5a). Additionally, in the 3D model of *DcTLR7* and *DcTOLL*, the three boxes were distinguished in different colors: red color (Box1), blue color (Box2) and orange color (Box3), respectively (Figure 5b).

### 3.7. The PPI Networks of DcTLRs

**Figure 6:**
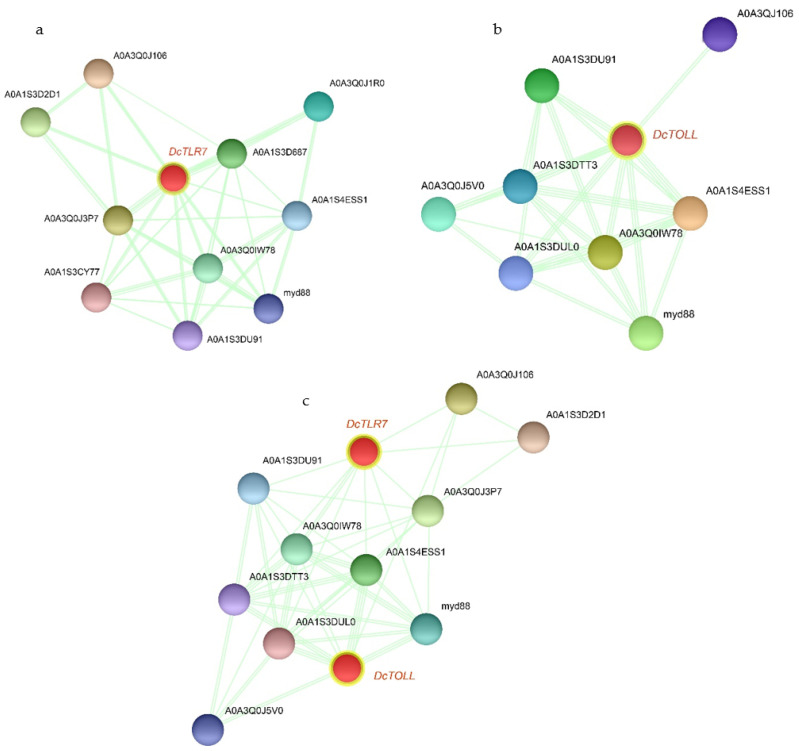
The PPI networks of *DcTLRs*. (**a**,**b**) The subnetworks *DcTOLL* and *DcTLR7*. (**c**) The interaction network of the two *DcTLRs*.

Based on the PPI network, *DcTLR7* and *DcTOLL* and their interacted proteins were shown in Figure 6a,b; *DcTLR7* possessed 11 interacting nodes, which showed the highest interaction; meanwhile, *DcTOLL* possessed 9 interacting nodes. There were 12 nodes in the interaction network of the two *DcTLRs* (Figure 6c). Most biological activities were regulated by protein-protein interaction; PPI analysis revealed that the signaling pathways were enriched significantly in factors including *myd88* (Myeloid differentiation primary response protein), *A0A1S3D2D1* (Immunoglobulin superfamily member 10), *A0A1S3D687* (Toll-like receptor Tollo; belongs to the Toll-like receptor family), *A0A1S4ESS1* (Embryonic polarity protein dorsal-like isoform X1), *A0A3Q0IW78* (Serine/threonine-protein kinase pelle-like isoform X1), *A0A1S3CY77* (TNF receptor-associated factor 4 isoform X1), *A0A3Q0J5V0* (Spaetzle-processing enzyme-like), *A0A1S3DTT3* (NF-kappa-B inhibitor cactus), *A0A1S3DUL0* (Nuclear factor NF-kappa-B p100 subunit). Importantly, *myd88* and *A0A1S4ESS1* were detected in the whole PPI network of the TLR families in *D. citri*.

### 3.8. Nymphs and Adults’ Specific Expression Profiles of DcTLRs When Fed on Endophytically Colonized C. sinensis Plants

**Figure 7:**
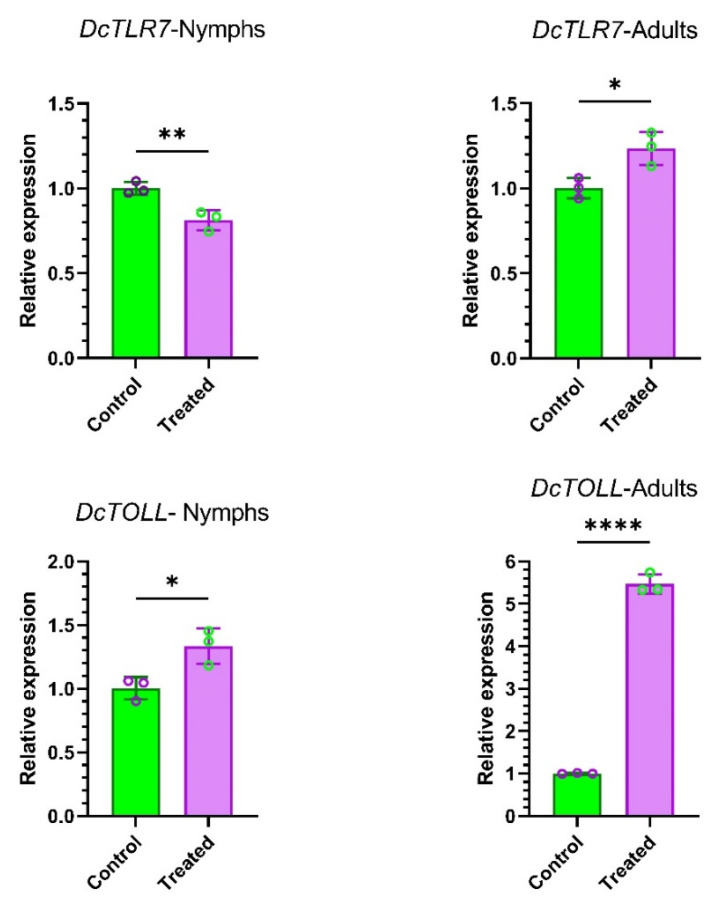
Expression profiles of *DcTLR* genes in nymphs and adults of *Diaphorina citri*. Mean ± SEM values were obtained from three biological replicates and three technical replicates. * *p* < 0.0332, ** *p* < 0.0021, **** *p* < 0.0001, Student’s *t*-test.

To understand the effects of endophytes in the expression patterns of *DcTLR* genes, we compared the relative expression of *DcTLRs* nymphs and adults when fed on endophyte-free plants (Control) vs endophytically colonized plans (Treated). The qRT-PCR results revealed that *DcTLR7* was weakly expressed compared with the control in nymphs; meanwhile, in adults it was highly expressed. On the other hand, *DcTOLL* was highly expressed in both nymphs and adults (Figure 7).

## 4. Discussion

TLRs conform a relatively complex family of innate immune genes that play a key role as the first line of defense against foreign pathogens in all multicellular organisms [36]. TLRs are the earliest described and most widely studied pattern recognition receptor in both vertebrates and invertebrates [37]. Toll-like receptors are well characterized in birds and mammals; however, they are currently lacking in the insect lineage and no effort has been made to systematically identify TLRs at the genome-wide level. Genome-wide analysis of TLR families has been carried out in a few arthropod species. For instance, a complete analysis has been performed for *M. domestica* (Diptera: Muscidae) [18]; as well, *TLR* families have been identified in a few aquatic species such as *Paralichthys olivaceus* (Pleuronectiformes: Paralichthyidae) [22], *Pelodiscus sinensis* (Testudines: Trionychidae) [38], *Litopenaeus vannamei* (Decapoda: Penaeidae), *Lateolabrax maculatus* (Perciformes: Lateolabracidae) [39], *Argyrosomus japonicus* (Acanthuriformes: Sciaenidae) [20]. However, the *D. citri* TLR gene family and their relevant immune responsiveness when fed on endophytically colonized plants have not yet been investigated. In this experiment, the systematic identification of TLRs’ family members has been completed in *D. citri* and their expression when fed on endophytically colonized and free-endophytes *C. sinensis* seedlings as rearing host.

The Asian citrus psyllid (ACP) *D. citri,* a common citrus plantation pest that is widely spread all over the world, possesses a potent and effective innate immune system that has helped develop insecticide resistance [40,41]. The bioinformatic analysis at the genome level revealed two *DcTLRs*, which were specified to be *DcTLR7* and *DcTOLL* based on the information of genome annotation and phylogenetic classification. Based on the determined *D. citri* TLR genes, we completed the analysis on the sequences, phylogeny, conserved domains, gene structure and gene expression profile under endophytically colonized and endophytes-free *C. sinensis* challenge seedlings to provide primarily comprehensive information about *DcTLRs* family members.

Our predicted results showed that *DcTLR7* and *DcTOLL* were located at the plasma membrane; similarly to our results, TLRs in mammals have been positioned at the same place [12,13]; likewise, the majority of TLRs of spotted sea bass are targeted in the plasma membrane [39]. *DcTLR7* contains 1323 residues, being the longest sequence, while *DcTOLL* contains only 374 residues; similarly to our results, Zhao, Wang, Li and Gai [18] reported that most *MdTLRs* contain >1000 residues, except for *MdTLR1*, which contains 860 residues. Secondary structural analysis showed that all *DcTLRs* are composed of two major domains, a leucine-rich repeat (LRR) domain in the extracellular region, a transmembrane region (TR), and a toll-interleukin receptor (TIR) in the intracellular region. A similar structure has been reported in mammals, teleost fish and other crustaceans [19]. According to this organization, Nie et al. [42] described that the LRR domain is important for pathogen recognition, while the intracellular TIR domain acts as the adaptor and initiates signaling. Additionally, the TLR gene analysis revealed a multiple extracellular LRR domain ranging from 3 (*DcTOLL*) to 23 (*DcTLT7*) which was almost similar to that described in previous studies, which had reported 5 to 25 LRR domains [18,19]. The fluctuation in the number of LRR domains detected in this study could be related to their response to diverse pathogens as the LRR is important for recognizing and binding ligands [43,44]. Therefore, we suggested that the recurrent number of LRR domains allows TLRs to identify a variety of pathogens.

The phylogenetic tree built in this study revealed that *DcTLR7* and *DcTOLL* clustered with those from *H. sapiens*, *M. musculus*, *B. mori*, *M. domestica*, *A. pisum*, *D. melanogaster* and *A. mellifera*. The relationship within these clusters shows the taxonomic location of these species during evolution, with a likely function in the immune response. The molecular weight of *DcTLRs* was higher. These results are consistent with those reported by Zhao, Wang, Li and Gai [18], who reported that the molecular weights of TLR in insects were significantly higher than the mammalians.

This work shows that when *D. citri* feeds on endophytically colonized *C. sinensis* seedlings, this leads to an enhancement of TLRs expression in both nymphs and adults of *D. citri*. The expression of *DcTOLL* was significantly enhanced compared with the control, and, similarly, the expression of *DcTLR7* significantly increased in both nymphs and adults when fed on endophytically colonized plants. These results are in line with those of a previous study that showed the infection of a pathogen induced the expression of TLRs [45]; however, in this study the expression was induced as result of insects feeding on endophytically colonized plants. It has been discovered that the downregulations or mutations of some extracellular receptors in insects, for example, GABA (gamma-aminobutyric acid), nACh (nicotinic acetylcholine receptor) receptor, APN (aminopeptidase N) and Cadherin, confer the resistance to synthetic insecticides [46,47]. Similarly, the activation of the TLRs leads not only to the responses and signaling but also binds extracellular chemicals or toxins [48]. Recently, it has been established that the arthropod Toll pathway plays a significant antibacterial function by monitoring the expression of immune-related genes [49,50].

According to Gao, et al. [51] the TIR domain interacts with the *myd88* adapter and mediates downstream immune signaling. Similarly, the PPI results elucidated that TLR genes interacted with immune-related mediating factors including interleukin-related genes, e.g., *myd88* dependent pathway, which could bind the TIR domain. According to Sugiyama et al. [52], *myd88* activates NF-kB via Toll-like receptor signaling. The *myd88* plays important role in immunity using its TIR domain networking with TLR and its N-terminal death domain networking with interleukin-1 receptor-associate kinase to trigger downstream signaling cascades and eliminate the invader [53]. Similarly, according to Takeda, Kaisho and Akira [48] the TLRs-induced inflammatory response is dependent on a common signaling pathway and is mediated by the adaptor molecule *myd88*; however, there is evidence for additional pathways that mediate TLR ligand-specific biological responses. Additionally, the structural alignments of different TIR domains from the different species selected demonstrated that they were highly conserved and had similar structural components and spatial arrangements. Similar structural components from different species have been reported by Zhao, Wang, Li and Gai [18]. This structural preservation of the TIR domain further ensures the functional and structural preservation of TLRs.

In summary, comprehensive analyses were performed and identified 2 *DcTLR* genes in the *D. citri* genome. The two identified *DcTLR* genes were subjected to analysis of physico-chemical features, phylogenetic classification, domain organization, structural alignments, chromosomal localization, protein-protein interaction and expression. Results from the expression analysis revealed that *B. bassiana* endophytically established as an endophyte in *C. sinensis* seedlings significantly upregulates *DcTLR* gene expression, activating the immune system of *D. citri*. Our present work provides systematic information and a comprehensive overview of the functional mechanisms and expression of *D. citri* TLRs in response to endophytes; additionally, the results provide a theorical foundation for further studies on the molecular mechanisms of TLRs in *D. citri*.

## Figures and Tables

**Table 1:** Characterization of identified TLR genes in *Diaphorina citri*.

Gene Name	Gene ID	ORF (bp)	Chr	Protein Length	CDS Length	MW (Da)	pI	GRAVY	Subcellular Location
*DcTLR7*	DcitrM093025.1.1	3972	ScVcwli_1258	1323	3972	150,543.47	6.09	−0.231	Plasma membrane
*DcTOLL*	DcitrM033265.1.1	1125	ScVcwli_3505	374	1125	43,503.94	6.01	−0.192	Plasma membrane

## Data Availability

The data used to support the findings of this study are included within the main text and supplementary files of this article.

## Supplementary Materials

The following supporting information can be downloaded at: https://www.mdpi.com/article/10.3390/jof8080888/s1, Table S1: Different species TLRs sequences, Table S2: Primer sequences, Table S3: First chain cDNA synthesis volume, Table S4: Real-time qPCR reaction volume.
