# Supplementary material for: Genome-Wide Identification and Characterization of Toll-like Receptors (TLRs) in Diaphorina citri and Their Expression Patterns Induced by the Endophyte Beauveria bassiana
Source: J Fungi (Basel). 2022 Aug 22;8(8):888. doi: 10.3390/jof8080888 (PMC9409752; doi:10.3390/jof8080888)
Supplement: Supplementary file 1 [file jof-08-00888-s001.zip › jof-1865252-supplementary.pdf]

Table S1. Different species TLRs sequences

Homo sapiens		*Mus musculus*		*Bombyx mori*		*Musca domestica*		*Acyrthosiphon pisum*		*Drosophila melanogaster*		*Apis mellifera*	
Gene name	Gene ID	Gene name	Gene ID	Gene name	Gene ID	Gene name	Gene ID	Gene name	Gene ID	Gene name	Gene ID	Gene name	Gene ID
HsTLR1	NP_003254	*MmTLR1*	NP_109607	*BmTLR3*	XP_012549875_1	*MdTLR1*	[illegible]01900185	*ApTOLL8*	XP_001948566_1	*DmTOLL1*	AAA28941	*AmTOLL*	XP_006562783_1
HsTLR2	NP_003255	*MmTLR2*	NP_036035	*BmTOLL6*	XP_012553339_1	*MdTLR2*	[illegible]01891253	*ApTOLL*	XP_016656770_1	*DmTOLL2*	AAF57509	*AmTOLL*	XP_016767399_1
HsTLR3	NP_003256	*MmTLR3*	NP_569054	*BmTOLL8*	XP_004921742_1	*MdTLR3*	[illegible]01892306	*ApTOLL*	XP_001942733_2	*DmTOLL3*	AAF86229	*AmTOLL*	XP_016767400_1
HsTLR4	NP_612564	*MmTLR4*	NP_067272	*BmTOLL*	XP_012546804_1	*MdTLR4*	[illegible]01889465	*ApTOLL*	XP_008182102_1	*DmTOLL4*	AAF52747	*AmTOLL*	XP_016767401_1
HsTLR5	NP_003259	*MmTLR5*	Q9JLF7	*BmTOLL2*	XP_004927316_1	*MdTLR5*	[illegible]01890408	*ApTOLL6*	XP_001947324_1	*DmTOLL5*	AAF86227	*AmTOLL*	XP_016767402_1
HsTLR6	NP_006059	*MmTLR6*	Q9EPW9	*BmTOLL3*	XP_021204430_1	*MdTLR6*	[illegible]01891471	*ApTOLL7*	XP_001946943_2	*DmTOLL6*	AAF86226	*AmTOLL*	XP_016767403_1
HsTLR7	NP_057646	*MmTLR7*	NP_573474	*BmTOLL7*	XP_004921732_1			*ApTOLL6*	XP_003248960_1	*DmTOLL7*	AAF57514	*AmTOLL*	XP_026295852_1
HsTLR8	NP_619542	*MmTLR8*	NP_573475	*BmTLR4*	XP_012546905_1					*DmTOLL8*	AAF86224	*AmTOLL*	XP_026295853_1
HsTLR9	NP_059138	*MmTLR9*	NP_112455	*BmTOLL7*	NP_001116821_1					*DmTOLL9*	AAF51581	*AmTOLL8*	XP_393713_3
HsTLR10	AF296673	*MmTLR11*	NP_991388	*BmTOLL7.2*	XP_004921727_1							*AmTOLL6*	XP_393712_2
		MmTLR12	NP_991392	*BmTOLL6.2*	XP_004921738_2							*AmTOLL7*	NP_001013379_1
		MmTLR13	NP_991389	*BmTOLL6.3*	XP_004921739_1							*AmTOLL8*	XP_016769909_1

Table S2. Primer sequences.

Prime mane	Sequence
ACT1	F: 5′- TGTGACGAAGAAGTTGCTGC -3′<br>R: 5′- TGGGGTATTTCAGGGTCAGG -3′
DcTLR7	F: 5′- TGAAGGACGGACTGGACATA -3′<br>R: 5′- ATACACCACATAGCCGTCG -3′
DcTOLL	F: 5′- CCCGTGTTATCATCCTGG -3′<br>R: 5′- GGTTCCTTGCCTGGAGATA-3′

Table S3. First chain cDNA synthesis volume.

Reactive	Volume
5× Fastking-RT SuperMix	4 μl
Total RNA	50 ng – 2 μg
RNase-Free ddH_2O	Make up to 20 μl

Reaction temperature	Reaction time
42℃	15 min
95℃	3min

Table S4. Real-time qPCR reaction volume.

Reactive	Volume
2× SuperReal PreMix Plus	10 μl
Forward primer	0.6 μl
Reverse primer	0.6 μl
cDNA	2.0 μl
RNase-Free ddH_2O	6.8 μl

Reaction temperature	Reaction time
95℃	15 min
95℃	10 sec
58℃	32 sec
Cycles	40 cycles
